# Association between pretreatment lymphocyte count and response to PD1 inhibitors in head and neck squamous cell carcinomas

**DOI:** 10.1186/s40425-018-0395-x

**Published:** 2018-08-31

**Authors:** Won Jin Ho, Mark Yarchoan, Alex Hopkins, Ranee Mehra, Stuart Grossman, Hyunseok Kang

**Affiliations:** The Sidney Kimmel Comprehensive Cancer Center at Johns Hopkins, 401 N Broadway, 1550 Orleans St., CRB2 5M44, Baltimore, MD 21287 USA

**Keywords:** Head and neck squamous cell cancers, Absolute lymphocyte count, NLR, PD1 inhibitor, Immunotherapy

## Abstract

**Background:**

Low absolute lymphocyte count (ALC) has previously been established as a marker of poor prognosis in multiple cancer types. There is growing evidence that ALC may also be associated with response to immunotherapy. This study explores whether response to PD1 inhibitors in recurrent and/or metastatic head and neck squamous cell carcinoma (R/M HNSCC) is associated with pretreatment ALC.

**Methods:**

Thirty-four R/M HNSCC patients who received either nivolumab or pembrolizumab between January 2014 and May 2018 at Johns Hopkins were identified retrospectively. Pretreatment blood counts in patients with and without clinical benefit from PD1 inhibitors were compared. Time-to-progression analyses were performed by dichotomizing the study cohort with the threshold of ALC 600 cells/μl, which is approximately 1.5 standard deviations away from treatment-naïve baseline mean.

**Results:**

Patients with lower ALC appeared to have significantly less clinical benefit from anti-PD1 therapy. Those patients with pretreatment ALC < 600 cells/μl also had shorter PFS than patients with pretreatment ALC ≥ 600 cells/μl (median PFS 60 days vs. 141 days, *p* < 0.05). These results were consistent with multivariate proportional hazards analyses demonstrating significant association with progression. These observations were further supported by an expansion cohort analysis incorporating additional fourteen R/M HNSCC patients who received other checkpoint immunotherapy regimens at our institution.

**Conclusions:**

This study for the first time demonstrates that pretreatment ALC is significantly associated with response to PD1 inhibitors in R/M HNSCC patients.

**Electronic supplementary material:**

The online version of this article (10.1186/s40425-018-0395-x) contains supplementary material, which is available to authorized users.

## Background

Immuno-oncology (IO) agents have revolutionized the treatment of cancers. Targeting the regulatory immune checkpoints, inhibitors of programmed cell death 1 (PD1) and cytotoxic T-lymphocyte-associated protein 4 (CTLA4) signaling have demonstrated efficacy and have been approved as standard therapeutic options in several cancer types [1]. In the management of recurrent or metastatic HNSCC (R/M HNSCC), pembrolizumab and nivolumab, both anti-PD1 antibodies, are now used as standard second line agents [2].

Anti-PD1 antibodies block the interaction of PD1 on CD8 T cells with the immune-suppressing ligand PD-L1 on antigen-presenting cells and tumor cells, thereby reversing immune tolerance [3]. Since the mechanism of anti-PD1 antibodies is thought to be dependent on the activity of functional T lymphocytes, it is rational to hypothesize that the efficacy of anti-PD1 antibodies would be compromised in patients with lymphopenia. To our knowledge, however, the impact of lymphocyte counts has not previously been investigated in a cohort of HNSCC patients on anti-PD1 therapies.

Importantly, growing evidence has demonstrated that conventional cancer treatment modalities may lead to significant lymphopenia [4–8]. Moreover, lymphopenia has long been associated with poorer prognosis in multiple cancer types [9–13]. This phenomenon is further highlighted in treatment regimens that involve protracted or fractionated radiation protocols such as pancreatic cancers [4], lung cancers [5], high grade gliomas [6], and HNSCCs [7]. The vast majority of patients with R/M HNSCC undergo a protracted course of chemoradiation leading to treatment-related lymphopenia, which is long-lasting. It is, however, unclear to date whether absolute lymphocyte counts (ALC) at the time of anti-PD1 therapy is correlated with responses to therapy in HNSCC patients. Similarly, increased neutrophil-to-lymphocyte ratio (NLR) has also been established as a poor prognostic factor in multiple cancer types including head and neck cancers [10, 14, 15]. While the ratio incorporates the neutrophil count in addition to the lymphocyte count, it is unclear whether the ratio provides any insight distinct from the lymphocyte count alone in the context of immunotherapy response.

In this single center retrospective review, we sought to determine whether baseline ALC prior to immunotherapy could be associated with immunotherapy response in R/M HNSCC. In parallel, we also explored whether pretreatment NLR provides additional data over ALC with regard to clinical outcomes.

## Methods

### Study design

Patients who received anti-PD1/PD-L1 antibodies either as a standard of care or on a clinical trial between January 2014 and May 2018 were identified retrospectively. Only patients who had been treated with single agent nivolumab or pembrolizumab in the recurrent/metastatic setting were included in the primary analysis. In an expanded cohort, patients who have been treated with other checkpoint inhibitors or combination regimens were also included. Patients who had prior exposure to immunotherapeutic agents, diagnosis of HIV leading to possible underlying immune dysfunction, or death within 4 weeks from the first dose of immune checkpoint inhibitor therapy were excluded from the analysis. The Institutional Review Board of Johns Hopkins Hospital approved this study (Johns Hopkins IRB00168156).

### Clinical parameters

For all patients, baseline Eastern Cooperative Oncology Group (ECOG) performance status at the time of initiating immunotherapy and records of prior lines of chemotherapy and radiation therapy were available for review. Baseline blood counts prior to immunotherapy was defined as the results obtained at the time (+/− 3 days) of initiating immunotherapy. Cell counts available at adjacent time points were reviewed to avoid selecting an outlier and to ensure that representative counts were recorded. Best treatment response was assessed by documented radiographic reports of complete response, partial response, stable disease, or progressive disease by Response Evaluation Criteria in Solid Tumors (RECIST) 1.1 as compared to the baseline imaging. Clinical benefit was defined as the best response of stable disease, partial response, or complete response. Progression of disease was defined by documentation of new clinical manifestation, new biopsy-proven disease, or clear radiographic progression. The imaging interval for most patients was every two to 3 months. For analyses involving dichotomization of the study cohort, we first determined the appropriate thresholds for ALC (and lymphocyte count as percentage of total white blood cells; L%), and NLR. To date, there are no widely accepted or standardized cutoffs for these parameters to be used as an associated marker of IO response. In prior studies looking at the overall prognosis, ALC 500–1500 cells/μl [7, 9, 11, 12], L% 8 [9], and NLR 2.5–5 [15, 16] have all been reported as thresholds. To establish statistically derived thresholds that also allow for adequate subcohort sizes for analysis, we referenced the treatment-naïve baseline means from our own cohort and selected the thresholds at approximately 1.5 standard deviations away from the means: ALC 600 cells/μl, L% 9, and NLR 7. Degrees of immune-related adverse events (irAEs) were graded by the Common Terminology Criteria for Adverse Events (CTCAE) v4.0. In analyzing the association between time from last radiation treatment and hematologic parameters, we previously observed that treatment-related lymphopenia can last over 12 months post-treatment and that approximately 50% of peripheral lymphocyte recovery seems to occur close to the 6-month follow up [7]. Thus, we compared the ALC, L%, and NLR values in subcohorts stratified by 180 days from the date of last radiation treatment.

### Statistical analysis

Chi-squared and Student’s t-test were used to compare patient characteristics. Student’s t-tests were also used to compare hematologic parameters between patient groups. Two-sided analysis was used for all t-tests. Progression free survival (PFS) was determined from the date of the first dose of IO agent and the date of true progression as evidenced by radiographic assessment or obvious clinical manifestation. Time-to-progression analyses were performed using the Kaplan-Meier method and Wilcoxon tests. Wilcoxon test was chosen over log-rank test, giving more weight to the earlier events, since most progression events occurred early in the follow-up. Hazard ratios were calculated by multivariate Cox-proportional hazards model with 95% confidence intervals (CI). *P* values less than 0.05 were considered statistically significant. All statistical analyses were performed using JMP Pro software (v.12; SAS institute, Cary, NC).

## Results

### Patient characteristics and overall clinical outcomes

Thirty-four R/M HNSCC patients received either nivolumab or pembrolizumab alone for recurrent or metastatic disease between January 2014 and May 2018 (Table 1). The most common primary site was the oropharynx (44.1%). Fifteen (41.2%) patients had HPV or p16 positive HNSCC, and five (14.7%) patients had EBV positive nasopharyngeal cancer. Sixteen of thirty-four patients (47.1%) had two or more lines of systemic therapy prior to the IO therapy including the first-line concurrent chemoradiotherapy (CRT) while the remaining eighteen patients had 0 or 1 prior lines of systemic therapy. Sixteen (47.1%) patients were treated with nivolumab and eighteen (52.9%) patients with pembrolizumab. Thirteen of the thirty-four patients in the cohort had ALC < 600 cells/μl at the time of starting IO therapy. The overall response rate (partial response or complete response) in the cohort was 32.3% and the median PFS was 3.2 months.

**Table 1:** Patient characteristics

	Number (%)	Number in those with clinical benefit (%)	Number in those without clinical benefit (%)	*P*
Gender				
Male	31 (91.2)	15 (88.2)	16 (94.1)	0.54
Female	3 (8.8)	2 (11.8)	1 (5.9)	
Age				
< 65	22 (64.7)	11 (17.7)	11 (17.7)	1.00
≥ 65	12 (35.3)	6 (35.3)	6 (35.3)	
Race				
White	25 (73.6)	11 (64.7)	14 (82.4)	0.49
Black	6 (17.7)	4 (23.5)	2 (11.8)	
Asian	2 (5.9)	1 (5.9)	1 (5.9)	
Hispanic	1 (2.9)	1 (5.9)	0 (0)	
Smoking history				
Yes	24 (70.6)	11 (64.7)	13 (76.5)	0.45
No	10 (29.4)	6 (35.3)	4 (23.5)	
ECOG				
0 or 1	31 (91.2)	15 (88.2)	16 (94.1)	0.54
≥ 2	3 (8.8)	2 (11.8)	1 (5.9)	
Primary tumor location				
Nasopharynx	5 (14.7)	4 (23.5)	1 (5.9)	0.51
Oral cavity	5 (14.7)	3 (17.6)	2 (11.8)	
Oropharynx	15 (44.1)	7 (41.2)	8 (47.1)	
Hypopharynx	1 (2.9)	0 (0)	1 (5.9)	
Larynx	6 (17.7)	2 (11.8)	4 (23.5)	
Sinus	2 (5.9)	1 (5.9)	1 (5.9)	
Virus association				
EBV	5 (14.7)	4 (23.5)	1 (5.9)	0.28
HPV/p16	14 (41.2)	7 (41.2)	7 (41.2)	
None	15 (44.1)	6 (35.3)	9 (52.9)	
Number of prior systemic therapy regimens				
0 or 1	18 (52.9)	11 (64.7)	7 (41.2)	0.17
2 or more	16 (47.1)	6 (35.3)	10 (58.8)	
PD-1 Inhibitor				
Nivolumab	16 (47.1)	9 (52.9)	7 (41.2)	0.49
Pembrolizumab	18 (52.9)	8 (47.1)	10 (58.8)	

*EBV* Epstein-Barr virus, *ECOG PS* Eastern Cooperative Oncology Group Performance Status, *HPV* human papillomavirus, *IO* Immuno-oncology

### Lymphopenia is associated with lack of clinical benefit to IO agents

**Fig. 1:**
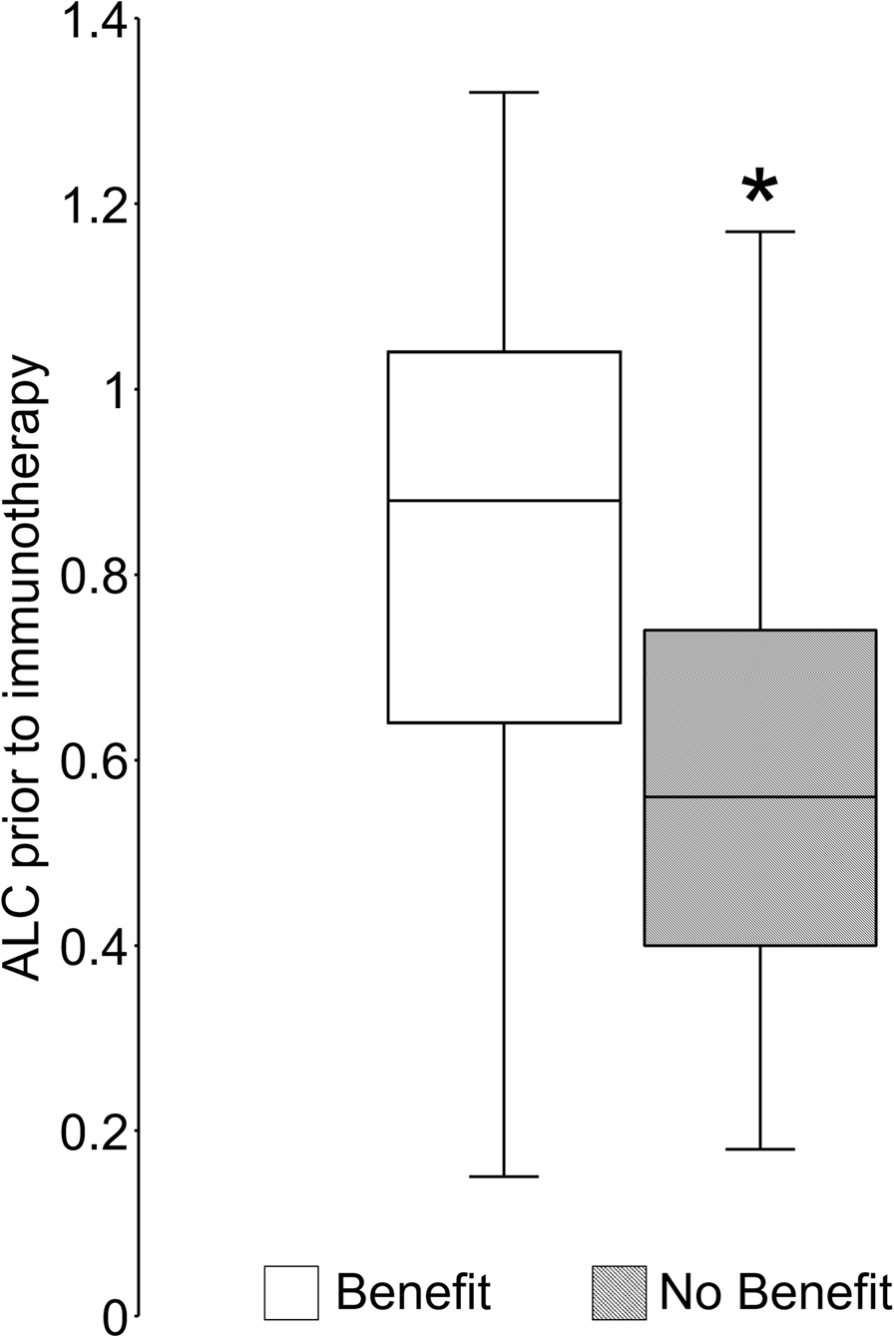
Absolute lymphocyte count (ALC) at the time of immunotherapy initiation was compared in patients who have demonstrated clinical benefit from pembrolizumab or nivolumab versus patients who have not. Pretreatment ALC was significantly lower in patients who have demonstrated lack of clinical benefit. Data is represented as box-and-whisker plot. *P < 0.05 by unpaired Student’s t-test

**Fig. 2:**
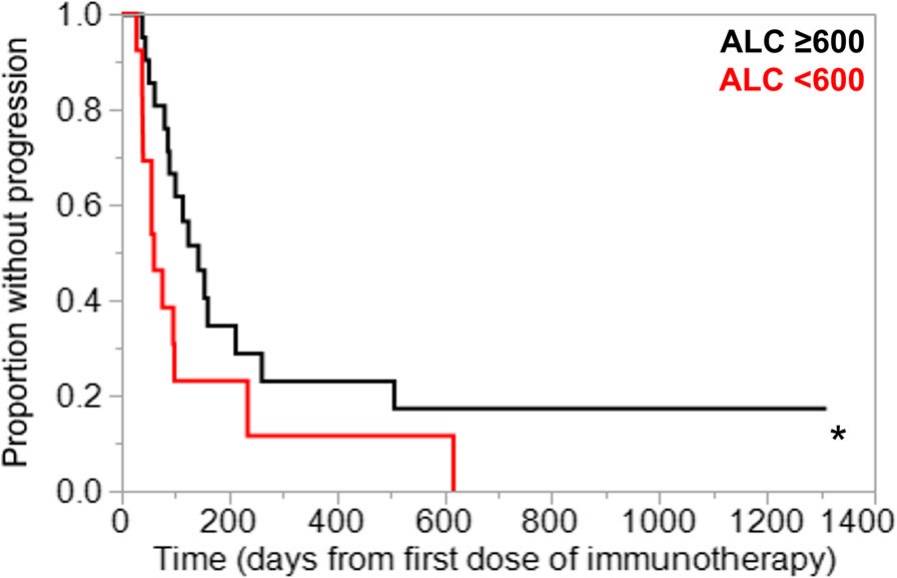
To compare Kaplan-Meier curves for time-to-progression analysis, the patient cohort treated with standard PD1 inhibitor therapy was stratified by absolute lymphocyte count (ALC) of 600 cells/μl as described in the methods section. Patients with ALC < 600 cells/μl were associated with significantly shorter PFS. *P < 0.05 by Wilcoxon test

**Fig. 3:**
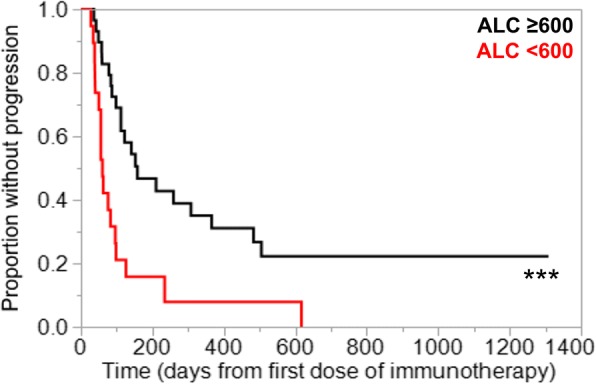
An expanded cohort time-to-progression analysis was performed by including additional patients who have received other checkpoint inhibitor regimens. Patients with ALC < 600 cells/μl were associated with significantly shorter PFS. ***P < 0.005 by Wilcoxon test

**Fig. 4:**
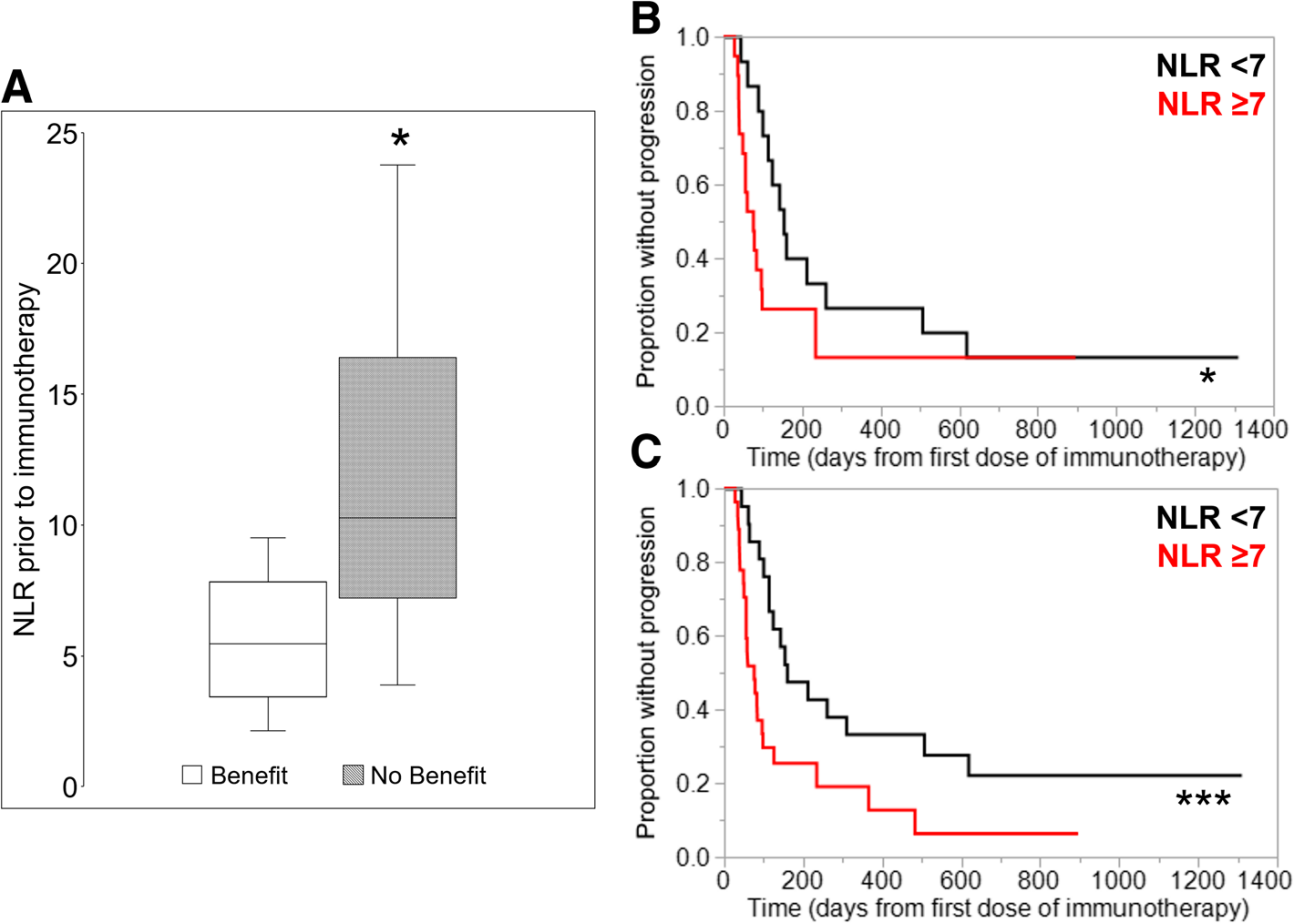
Associations between pretreatment NLR and clinical outcomes were analyzed. **a** NLR at the time of immunotherapy initiation was compared in patients who have demonstrated clinical benefit from pembrolizumab or nivolumab versus patients who have not. Pretreatment NLR was significantly higher in patients who have demonstrated lack of clinical benefit. Data is represented as box-and-whisker plot. *P < 0.05 by unpaired Student’s t-test. To compare Kaplan-Meier curves for time-to-progression analysis, the patient cohorts were stratified by NLR of 7 as described in the methods section. Patients with NLR ≥ 7 were associated with significantly shorter PFS in both the (**b**) primary cohort analysis and (**c**) expanded cohort analysis. *P < 0.05, ***P < 0.005 by Wilcoxon test

In the primary analysis of thirty-four patients treated with either nivolumab or pembrolizumab alone, lower ALC was significantly associated with lack of clinical benefit (Fig. 1). Also, ALC < 600 cells/μl was associated with shorter PFS (median 60 days vs. 141 days, *p* = 0.03; Fig. 2). As a further proof-of-concept, the cohort was expanded to include fourteen additional R/M HNSCC patients who received other anti-PD1/PD-L1 antibody-based combination IO regimens such as anti-CTLA4 antibody or anti-KIR antibody (Additional file 1: Table S1). Based on this expanded cohort, similar results were observed with even greater statistical significance, demonstrating that lymphopenia was associated with poorer response to IO therapy in HNSCC patients (median 60 days vs. 158 days, *p* = 0.0006; Fig. 3). Results from multivariate proportional hazards analyses were also consistent with these findings for patients with ALC < 600 cells/μl, demonstrating hazard ratios of 3.65 in the primary cohort analysis (*p* = 0.04; Table 2) and 4.89 in the expanded cohort analysis (*p* = 0.001; Table 2). Consistent with ALC associations, lower pretreatment L% was also significantly associated with poorer clinical outcomes (Additional file 1: Figure S1, Table 2). When performing the same analysis with pretreatment NLR in both primary and expanded cohorts, increased NLR was associated with lack of clinical benefit (Fig. 4a), shorter PFS (Fig. 4b, c), and significant hazard ratios (Table 2). Neither primary nor expanded cohort demonstrated significant interaction between clinical benefit and the site of disease, locoregional versus distant.

**Table 2:** Multivariate proportional hazard ratios

Parameter	Hazard Ratio	Lower 95%	Upper 95%	*P* value
Primary Cohort				
ALC < 600 vs. ≥600	3.65	1.05	12.98	0.04
L% < 9 vs. ≥9	9.68	2.91	37.62	0.0002
NLR ≥7 vs. < 7	14.74	4.14	63.96	< 0.0001
Expanded Cohort				
ALC < 600 vs. ≥600	4.89	1.90	12.90	0.001
L% < 9 vs. ≥9	5.55	2.13	15.72	0.0004
NLR ≥7 vs. < 7	4.69	1.99	11.72	0.0004

Multivariate analyses were adjusted for the following independent variables: age, gender, race, smoking history, primary site of cancer, viral association, ECOG performance status, number of prior lines of therapy, and immunotherapy regimen; *ALC* Absolute lymphocyte count, *L%* Lymphocyte %, *NLR* Neutrophil-lymphocyte ratio

### Immune-related adverse events

Eight of thirty-four (23.5%) patients receiving nivolumab or pembrolizumab developed immune-related adverse events (irAEs) of any grade, and three of thirty-four (8.8%) of the patients developed grade 3 or 4 irAEs (Table 3). A total of three patients developed pneumonitis and thyroiditis, and two patients developed colitis. Five of the eight patients with any grade irAE and two of the three patients with grade 3 or 4 irAEs had pretreatment ALC ≥ 600 cells/μl. The number of these events were inadequate for statistical analyses.

**Table 3:** Details of immune-related adverse events (AEs) in the study cohort

Immune-related AEs	Any grade (# of patients)	Any grade (% of patients)	Grade 3 or 4 (# of patients)	Grade 3 or 4 (% of patients)
All types	8^a^	23.5%^a^	3	8.8%
Dermatitis – Rash	1	2.9%	0	0
Colitis	2	5.9%	0	0
Thyroiditis	3	8.8%	0	0
Pneumonitis	3	8.8%	2	5.9%
Thrombocytopenia	1	2.9%	0	0
Others				
Autonomic dysregulation	1	2.9%	1	2.9%
Renal graft rejection	1	2.9%	N/A	N/A

^a^Some patients developed more than one type of immune-related AEs

### Concurrent chemoradiotherapy is associated with lymphopenia

In a subcohort of twenty-one patients who had undergone CRT and for whom all of the necessary records were available, we confirmed that CRT leads to significantly decreased absolute lymphocyte count (Additional file 1: Figure S2). CRT-related lymphopenia was accompanied by overall leukopenia, neutropenia, decreased percent of lymphocytes, and increased NLR (Additional file 1: Figure S2). When comparing the counts from before and after CRT, patients dropped their ALCs from the treatment-naïve baseline mean of 1400 cells/μl to mean of 300 cells/μl (*p* < 0.0001; Additional file 1: Figure S1B).

### Exploring potential factors that impact lymphopenia and increased NLR

Finally, we sought to explore what may be the important clinical parameters that influence ALC and NLR. Two most intuitive parameters interrogated in this study were duration from last radiation therapy and the number of prior lines of systemic therapy. Patients who have received radiation within the past 180 days had significantly lower ALC, lower L%, and higher NLR (Additional file 1: Figure S3). However, there were no significant associations between the number of prior lines of systemic therapy and ALC, L%, or NLR (Additional file 1: Figure S4).

## Discussion

Previously, we observed that CRT leads to development of significant lymphopenia in HNSCC patients [7]. In this study, we confirmed again that CRT indeed leads to lymphopenia in HNSCC patients. We further expanded this observation to show that CRT leads not only to lymphopenia but also overall leukopenia, neutropenia, decreased percent of lymphocytes, and increased NLR. We have also previously shown that lymphopenia is closely linked with prior radiation but not necessarily prior systemic chemotherapy [5]. Consistent with this, we observed in this HNSCC cohort that duration from last radiation treatment was a significant factor associated with pre-IO lymphopenia. In contrast, the number of prior systemic lines of therapy did not significantly associate with pre-IO lymphopenia although this lack of significance may be explained by the heterogeneity of the systemic regimens in terms of drugs, cumulative dose, and timing.

Importantly, low ALC was significantly associated with lack of clinical benefit from IO therapy; R/M HNSCC patients with low ALC < 600 cells/μl were associated with significantly worse response to therapy and shorter PFS. Patients in the lower ALC group had median PFS of 2 months, which mirrored those of previous studies [17–19]. Interestingly, in the higher ALC group, the median PFS was approximately 5 months. Overall, PD-1 inhibitor therapy in this study appeared to demonstrate superior ORR compared to previous studies [17–19].

To our knowledge, this study for the first time demonstrates a significant association between pretreatment lymphocyte counts and IO therapy response in a cohort of R/M HNSCC patients. Our observations provide additional evidence that the peripheral lymphocyte count may be an important parameter to consider when administering anti-PD1 therapy. These findings are particularly critical for three fundamental reasons: (i) the vast majority of locally advanced HNSCC patients will develop lymphopenia due to the first-line CRT, (ii) immunotherapy has now become standard second-line therapeutic option for patients with R/M HNSCC, and (iii) we currently do not have a way to predict which patients will respond to IO therapy. Predictive biomarkers of PD1 inhibitor therapy other than PD-L1 expression, which is of limited utility in HNSCCs, are much in need, and this study gives further support to the potential utility of ALC as such biomarkers. Mechanistically, even though the total lymphocyte count alone is far from fully representing the state of antitumor immune response, one can speculate that it may be a surrogate indicator of lymphocyte presence in the tumor microenvironment and/or the degree of immunosurveillance.

In this study, we had the opportunity to evaluate whether the incorporation of neutrophil counts into a ratio against lymphocyte counts provided any unique insight into immunosuppression in this population that might have been missed if just ALC was evaluated. In fact, the use of NLR did not provide any additional insight. Although studies have suggested the importance of tumor-associated neutrophils in pro-tumorigenic processes [20], any potential association between the absolute neutrophil counts and response to anti-PD1 therapy remains largely uncharacterized and of unclear importance. Furthermore, neutrophil counts recover more readily from prior RT compared to lymphocyte counts and may be drastically influenced by various clinical contexts, e.g. active infections or acute glucocorticoid use, confounding the utility of NLR. Additional studies are necessary to determine whether ALC is the more reliable correlate for anti-PD1 therapy response.

In cancer types other than HNSCC, prior studies have shown that pretreatment lymphocyte counts as well as NLR are potential biomarkers associated with response to IO therapy [21, 22]. Several groups have observed that ALC and/or NLR are associated with response to IO therapy in melanoma patients [14, 23–25]. We have also previously reported a significant association between these parameters and IO therapy response in a heavily heterogeneous cohort of multiple cancer types, e.g. lung, melanoma, renal cell, urothelial, and HNSCC (eight of 167 patients) [26]. In HNSCC patients, there has been a recent report showing a significant association between high NLR (> 8.77) and poorer clinical outcomes with anti-PD1 therapy [27].

Limitations of the study include both the retrospective and single-center nature of the study as well as the small size of the cohort. Also, the current cohort represented a relatively more treatment-naïve group of patients as suggested by the apparently higher ORR and longer overall PFS compared to previous studies [17–19]. Another limitation may be the inclusion of some patients with nasopharyngeal carcinoma (NPC), which was not included in landmark trials that have led to the approval of anti-PD1 antibodies in HNSCC; the use of pembrolizumab in NPCs is currently a category 2B recommendation based on the latest update of the National Comprehensive Cancer Network guidelines [2]. Additionally, it cannot be determined from our data whether lymphopenia is a truly predictive biomarker of anti-PD1 immunotherapy, or simply a global prognostic biomarker that reflects an advanced disease state irrespective of treatment intervention. Lastly, other potential prognostic markers of IO response, e.g. PDL1 expression and tumor mutational burden for non-virally associated tumors, could not be factored into the multivariate analyses.

A critical next step to confirming the utility of these correlates as predictive biomarkers of IO therapy response would be to confirm the observations in a prospective trial. It would also be valuable to establish the biologic significance and the mechanism behind peripheral lymphocyte counts and IO therapy response, e.g. whether peripheral lymphocyte counts truly correlate with the compositional changes in the tumor microenvironment and/or functional changes in the antitumor immunity, specifically in the context of CRT-associated lymphopenia. To venture further beyond these ideas, a challenging task would be to determine whether allowing for treatment-related lymphopenia to recover, when done in appropriate clinical contexts, in fact improves the response to IO therapy in the patients who actually recover. Thus, it would be interesting to explore whether treatment strategies aimed at reducing treatment/radiation-related lymphopenia in HNSCC patients would truly lead to superior clinical outcomes.

## Conclusions

In conclusion, this study demonstrates for the first time that pretreatment ALC in R/M HNSCC patients is significantly associated with response to PD1 inhibitor therapy and offers a potential predictive biomarker to be explored further.

## Additional file

Additional file 1: **Figure S1.** Associations between pretreatment L% and clinical outcomes. **Figure S2.** Hematologic parameters in the peripheral blood of HNSCC patients were compared between before concurrent chemoradiotherapy (CCRT) and at the time of CCRT completion. **Figure S3.** Associations between absolute lymphocyte count (ALC; A), lymphocyte percentage (L%; B), neutrophil-to-lymphocyte ratio (NLR; C) at the time of immunotherapy initiation and previous RT within the past 180 days. **Figure S4.** Associations between absolute lymphocyte count (ALC; A), lymphocyte percentage (L%; B), neutrophil-to-lymphocyte ratio (NLR; C) at the time of immunotherapy initiation and number of prior lines of systemic therapy (up to 1 versus 2 or more). **Table S1.** Patient characteristics in the expanded cohort. (DOCX 1002 kb)

## Abbreviations

ALC: Absolute Lymphocyte Count
ANC: Absolute Neutrophil Count
CTCAE: Common Terminology Criteria for Adverse Events
CTLA-4: Cytotoxic T-Lymphocyte Activation Protein 4
EBV: Ebstein-Barr Virus
ECOG PS: Eastern Cooperative Oncology Group Performance Status
HPV: Human Papillomavirus
IO: Immuno-Oncology
irAE: Immune-related Adverse Event
L%: Lymphocyte Percentage (of Total WBC)
NLR: Neutrophil-to-Lymphocyte Ratio
NPC: Nasopharyngeal Carcinoma
PD1: Programmed Cell Death Protein 1
PD-L1: Programmed Death-Ligand 1
PFS: Progression-Free Survival
R/M HNSCC: Recurrent and/or Metastatic Head and Neck Squamous Cell Carcinoma
RECIST: Response Evaluation Criteria in Solid Tumors
WBC: White Blood Cell Count
