# Supplementary material for: Association between pretreatment lymphocyte count and response to PD1 inhibitors in head and neck squamous cell carcinomas
Source: J Immunother Cancer. 2018 Aug 31;6:84. doi: 10.1186/s40425-018-0395-x (PMC6117944; doi:10.1186/s40425-018-0395-x)
Supplement: Supplementary file 1 — Figure S1. Associations between pretreatment L% and clinical outcomes. Figure S2. Hematologic parameters in the peripheral blood of HNSCC patients were compared between before concurrent chemoradiotherapy (CCRT) and at the time of CCRT completion. Figure S3. Associations between absolute lymphocyte count (ALC; A), lymphocyte percentage (L%; B), neutrophil-to-lymphocyte ratio (NLR; C) at the time of immunotherapy initiation and previous RT within the past 180 days. Figure S4. Associations between absolute lymphocyte count (ALC; A), lymphocyte percentage (L%; B), neutrophil-to-lymphocyte ratio (NLR; C) at the time of immunotherapy initiation and number of prior lines of systemic therapy (up to 1 versus 2 or more). Table S1. Patient characteristics in the expanded cohort. (DOCX 1002 kb) [file 40425_2018_395_MOESM1_ESM.docx]

Additional file 1


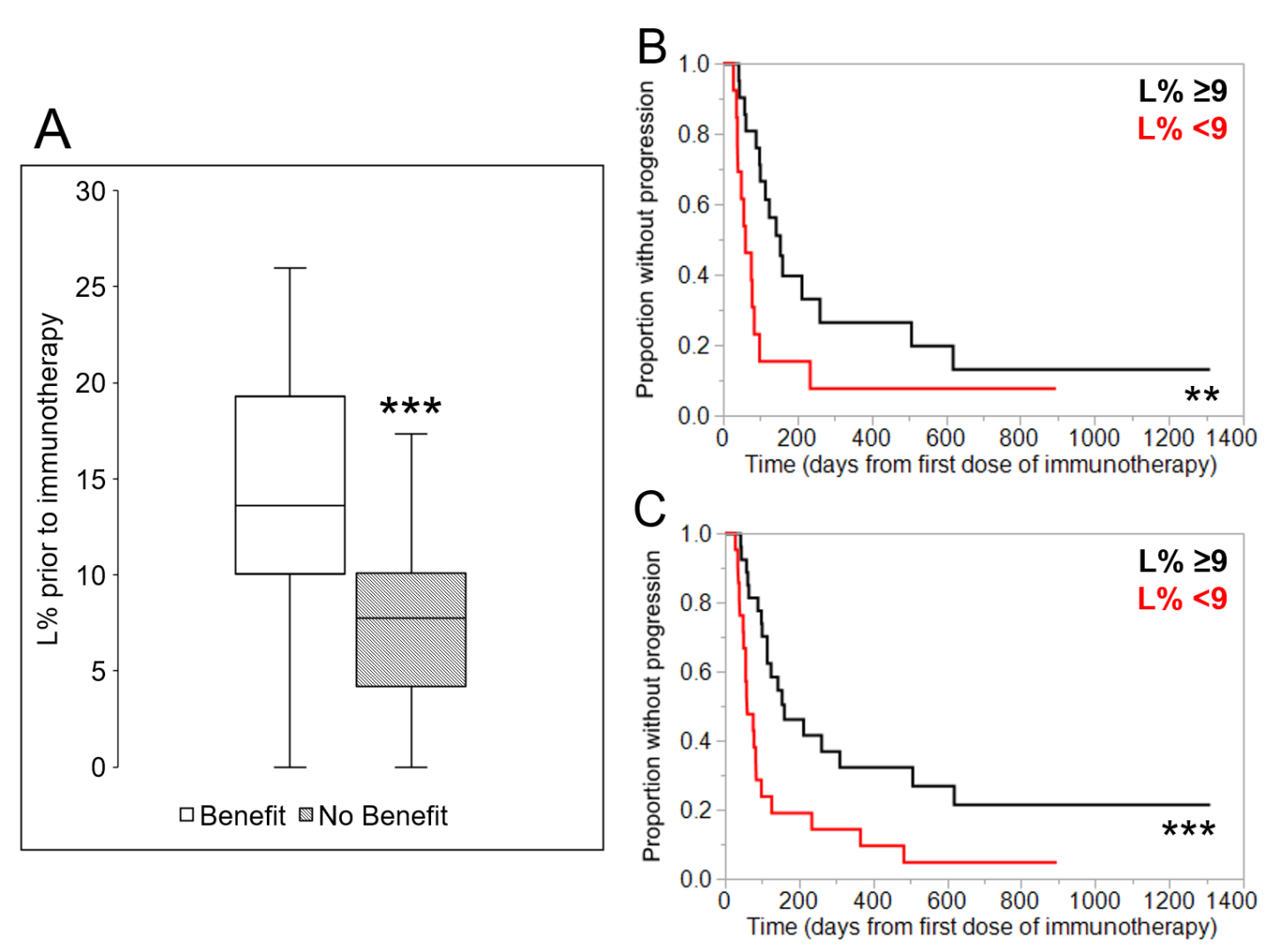


**Figure S1**

Associations between pretreatment L% and clinical outcomes were analyzed. (A) L% at the time of immunotherapy initiation was compared in patients who have demonstrated clinical benefit from pembrolizumab or nivolumab versus patients who have not. Pretreatment L% was significantly lower in patients who have demonstrated lack of clinical benefit. Data is represented as box-and-whisker plot. ***P<0.005 by unpaired Student’s t-test. To compare Kaplan-Meier curves for time-to-progression analysis, the patient cohorts were stratified by L% of 9 as described in the methods section. Patients with L%<9 were associated with significantly shorter PFS in both the (B) primary cohort analysis and (C) expanded cohort analysis. **P<0.01, ***P<0.005 by Wilcoxon test.

**Figure S2**

**
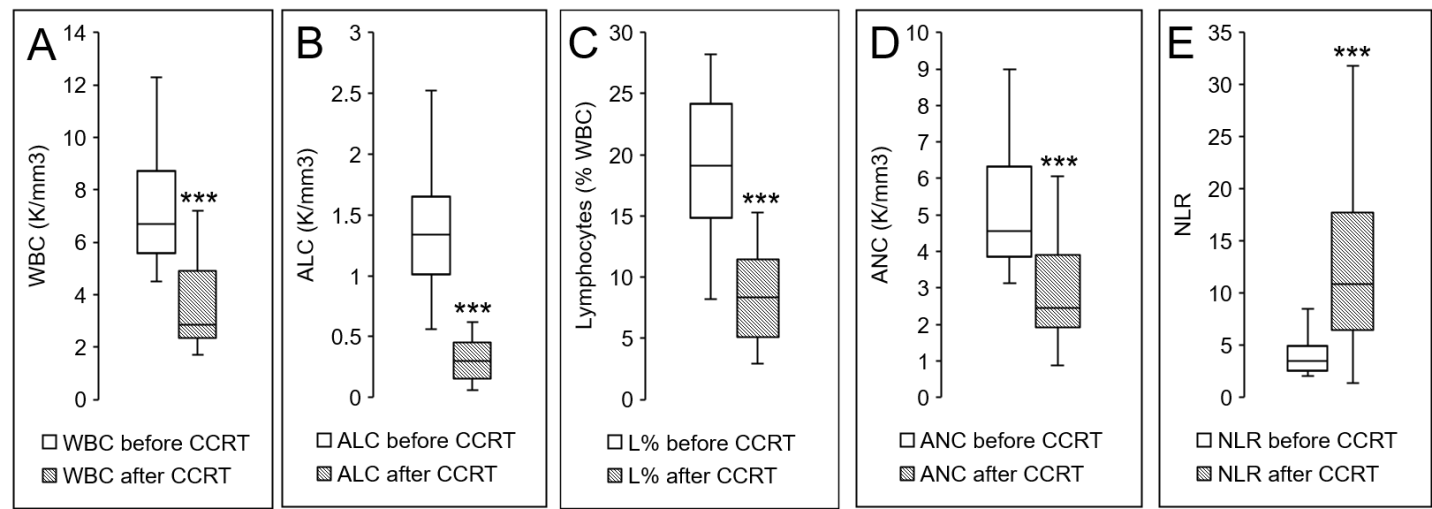
**

Hematologic parameters in the peripheral blood of HNSCC patients were compared between before concurrent chemoradiotherapy (CCRT) and at the time of CCRT completion. Total white blood cell counts (WBC; A), absolute lymphocyte count (ALC; B), lymphocyte percentage (L%; C), and absolute neutrophil count (ANC; D) all decreased significantly with CCRT. Neutrophil-to-lymphocyte ratio (NLR; E) increased significantly with CCRT. Data is represented as box-and-whisker plot. ***P<0.005 by paired Student’s t-test.

**Figure S3**


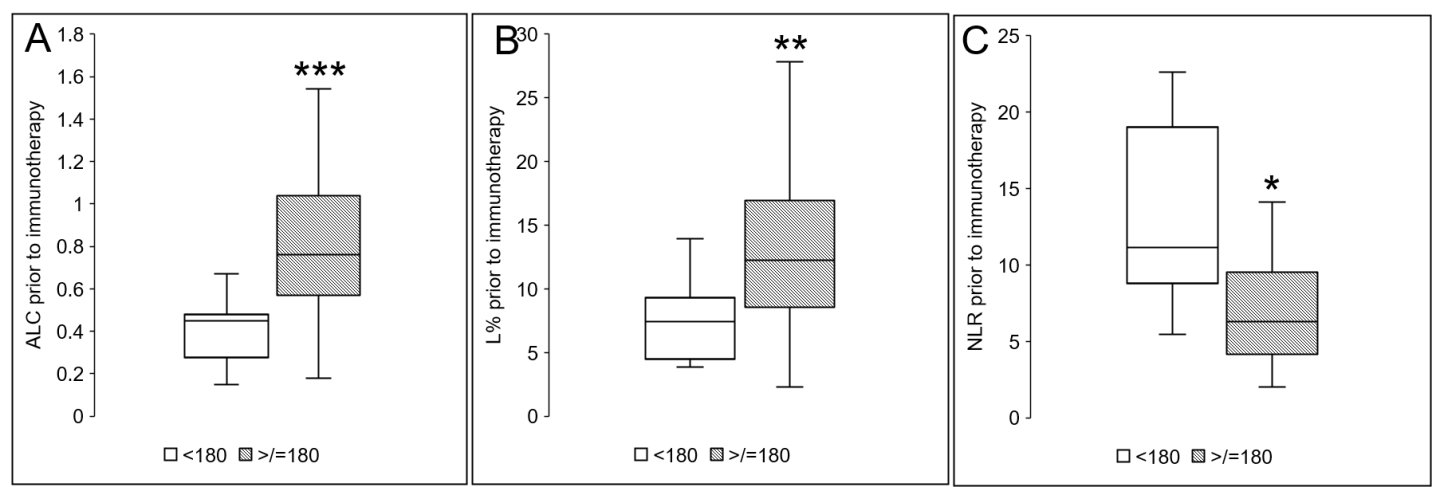


To determine whether time from last radiation therapy (RT) is significantly associated with pretreatment hematologic parameters, absolute lymphocyte count (ALC; A), lymphocyte percentage (L%; B), and neutrophil-to-lymphocyte ratio (NLR; C) at the time of immunotherapy initiation were compared in patients who have had any RT for any indication within the past 180 days and patients who have not had last RT within the past 180 days. Patients who have had any RT within the past 180 days had significantly lower ALC, L%, and NLR. Data is represented as box-and-whisker plot. *P<0.05, **P<0.01, ***P<0.005 by unpaired Student’s t-test.

**Figure S4**


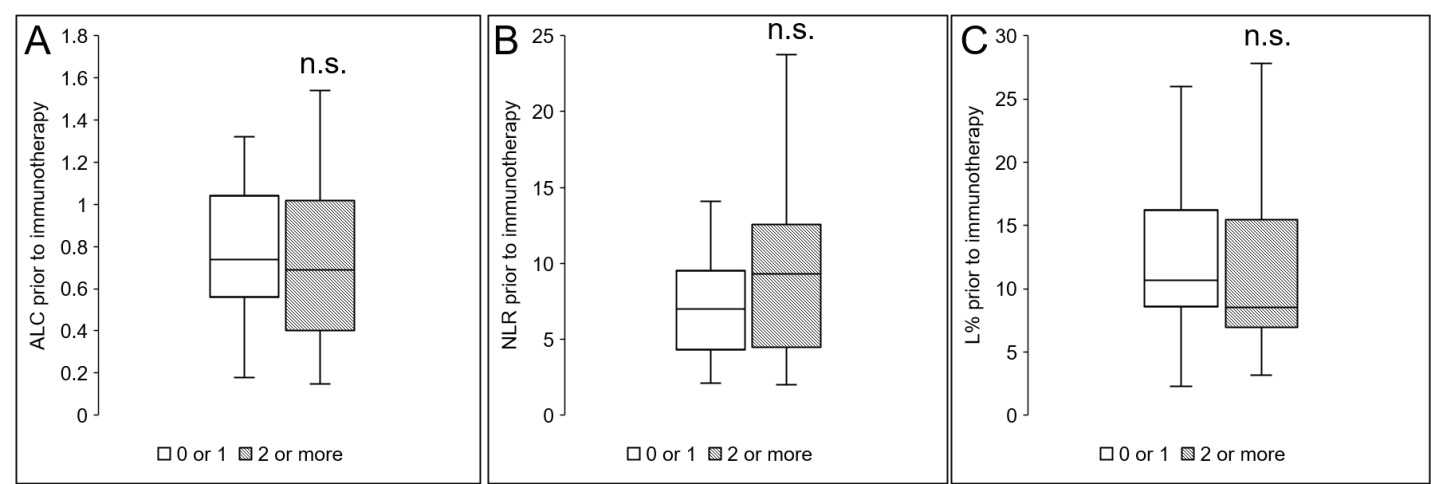


To determine whether number of prior lines of systemic therapy is significantly associated with pretreatment hematologic parameters, absolute lymphocyte count (ALC; A), lymphocyte percentage (L%; B), and neutrophil-to-lymphocyte ratio (NLR; C) at the time of immunotherapy initiation were compared in patients who have had 0 or 1 prior lines and patients who have had 2 or more prior lines of any systemic therapy. Pretreatment ALC, L%, and NLR were not significantly different in these groups. Data is represented as box-and-whisker plot. Not significant (n.s.) by unpaired Student’s t-test.

**Table S1. Patient characteristics in the expanded cohort**

|  | Number (%) | Number in those with clinical benefit (%) | Number in those without clinical benefit (%) | P |
| --- | --- | --- | --- | --- |
| Gender  Male  Female | 41 (85.4)  7 (14.6) | 21 (84.0)  4 (16.0) | 20 (87.0)  3 (13.0) | 0.77 |
| Age  <65  ≥65 | 31 (64.6)  17 (35.4) | 16 (64.0)  9 (36.0) | 15 (65.2)  8 (34.8) | 0.93 |
| Race  White  Black  Asian  Hispanic | 35 (72.9)  9 (18.8)  3 (6.3)  1 (2.1) | 17 (68.0)  6 (24.0)  1 (4.0)  1 (4.0) | 18 (78.3)  3 (13.0)  2 (8.7)  0 (0) | 0.44 |
| Smoking history  Yes  No | 31 (64.6)  17 (35.4) | 14 (56.0)  11 (44.0) | 17 (73.9)  6 (26.1) | 0.19 |
| ECOG PS  0 or 1  ≥2 | 45 (93.8)  3 (6.3) | 23 (92.0)  2 (8.0) | 22 (95.7)  1 (4.4) | 0.60 |
| Primary tumor location  Nasopharynx  Oral cavity  Oropharynx  Hypopharynx  Larynx  Sinus | 5 (10.4)  8 (16.7)  24 (50.0)  1 (2.1)  8 (16.7)  2 (4.2) | 4 (16.0)  3 (12.0)  13 (52.0)  0 (0)  4 (16.0)  1 (4.0) | 1 (4.4)  5 (21.7)  11 (47.8)  1 (4.4)  4 (17.4)  1 (4.4) | 0.56 |
| Virus association  EBV  HPV/p16  None | 5 (10.4)  21 (43.8)  22 (45.8) | 4 (16.0)  12 (48.0)  9 (36.0) | 1 (4.4)  9 (39.1)  13 (56.5) | 0.22 |
| Number of prior systemic therapy regimens  0 or 1  2 or more | 25 (52.1)  23 (47.9) | 14 (56.0)  11 (44.0) | 11 (47.8)  12 (52.2) | 0.57 |
| Immunotherapy regimen  Pembrolizumab  Nivolumab  Nivolumab + another IO agent  Other IO regimens | 18 (37.5)  16 (33.3)  12 (25.0)  2 (4.2) | 8 (32.0)  9 (36.0)  7 (28.0)  1 (4.0) | 10 (43.5)  7 (30.4)  5 (21.7)  1 (4.4) | 0.87 |

EBV, Epstein-Barr virus; ECOG PS, Eastern Cooperative Oncology Group Performance Status; HPV, human papillomavirus; IO, immuno-oncology
